# ‘Athletes’, ‘Talents’, and ‘Players’: Conceptual Distinctions and Considerations for Researchers and Practitioners

**DOI:** 10.1007/s40279-024-02101-5

**Published:** 2024-08-29

**Authors:** Adam Grainger, Adam L. Kelly, Stephen W. Garland, Joseph Baker, Kathryn Johnston, Alexander B. T. McAuley

**Affiliations:** 1 Kitman Labs, Dublin, Ireland; 2 Institute of Sport and Health, University College Dublin, Belfield, Dubin 4, Ireland; 3 Ulster Rugby, Belfast, Northern Ireland; 4 Research for Athlete and Youth Sport Development (RAYSD) Lab, Centre for Life and Sport Sciences (CLaSS), College of Life Sciences, Birmingham City University, Birmingham, UK; 5 Department of Sports Sciences, Malmö University, Malmö, Sweden; 6 Tanenbaum Institute for Science in Sport, University of Toronto, Toronto, ON Canada

## Abstract

A clearer understanding of, and tighter boundaries between, terms are important for researchers designing studies as well as for other sport stakeholders creating evidence-informed policies. This article considers the terms ‘athlete’, ‘talent’, and ‘player’ from psychological and sociocultural perspectives and in different sporting communities to highlight the importance of terminological clarity in sport research. We present considerations to clarify the use of these terms within different contexts and how the use of specific terms may affect knowledge mobilization in diverse sporting populations. A conceptual discussion is provided to help operationalize development-related terminology and its associated stages, to better reflect contemporary academic thought, and enhance practical interpretations. Importantly, we also call for greater transparency from researchers when presenting findings and encourage practitioners to clearly define key terms when working in sport. Our intention in this paper is to energize readers to consider how we use language in athlete identification and development contexts, to stimulate deeper thought and discourse around the possible implications these terms may have at any point of an individual’s development in sport. Greater deliberation, identification, and acknowledgment of the drawbacks accompanying these terms will be needed before more confident assertions can be made on how researchers and practitioners could (or even should) implement certain terminology across youth sport contexts moving forward. This paper adds to a growing literature on the importance of clarity in terminology and acts as an impetus for those working in specific sports to co-design key terms used by researchers, practitioners, and policy makers.

## Key Points

**Table:** 

Inconsistencies in the use of certain words in relation to athlete development are common.
This paper adds to a growing literature on the importance of terminological clarity.
In particular, this paper advocates that researchers and practitioners working in specific sports clearly detail how they are using terms such as athlete, talent, and player.

## Introduction

Effective application of scientific results to those who participate in sport and exercise requires a clear understanding of the context of the research (e.g., study design, participant characteristics) and how the results apply therein (e.g., analysis and interpretation). Underpinning this is a requirement for a common understanding of the terms and concepts used, along with their consistent application. While inconsistency may not always cause confusion because the sender and receiver of the message could be consistent with one another (i.e., they have the same understanding), it is unwise to assume this is always the case, especially in research when definitional clarity is imperative for minimizing misinterpretations and maximizing performance outcomes. Indeed, such misunderstandings could result in the misuse of resources, poor alignment of organizational priorities, or inefficient decision making, among other consequences. Resources such as time, funding, and facilities are limited, and most competitive sport organizations rely on precision and accuracy in pursuit of strategic goals. As a result, it is crucial that those involved in knowledge creation, sharing, and application (e.g., researchers, practitioners, and policy makers) consider the ways in which key terms are defined and used across time and space [1]. In particular, sport scientists should strive to use words as clearly, consistently, and objectively as possible [2].

Inconsistencies in the use of certain words in relation to athlete development [3], where the same word is used in different ways, or a variety of words are used to refer to the same thing, make it difficult to be sure to whom the results can be applied. For instance, the term “elite” has been used in a wide variety of sporting contexts, from under nine age groups to senior international levels as well as being inferred based on accumulated training and general experience [3]. The sections below consider some words that are commonly misconstrued and misinterpreted in sport, words describing those who participate in sport (i.e., organized individual or team activities), and examine their use across different contexts and phases of development.

## Athlete, Athleticism, and Athletic

We first consider perhaps the most common descriptor of someone who participates in sport—an ‘athlete’. Dictionary definitions of ‘athlete’ vary from “a person who is very good at sports or physical exercise, especially one who competes in organized events” [4] to “an athlete is a person who does a sport, especially athletics, or track and field events” [5]. As noted, the term is used to describe a wide spectrum of the physically active population (i.e., those who participate in sport) and not just those who are proficient in a specific domain [2]. There are also different types of athletes described in the extant literature—recreational athletes, university/collegiate athletes, elite athletes, competitive athletes, and talented athletes, amongst others. These attributive adjectives can also be blurry and overlapping; tighter boundaries could be defined for such terms, and may improve research designs and knowledge uptake. For example, when does a recreational athlete become competitive? Or can that person be both at the same time?

It could be argued that to be considered an athlete, one must demonstrate athleticism (i.e., the combination of qualities such as speed, strength, and agility; [6]) and be athletic (i.e., physically strong and active, good at sports [7]). While these may appear intuitive, the classification and measurement of such terms are not clear. In a recent study by Till et al. [8], for example, practitioners perceived definitions of athleticism and long-term athletic development inconsistently. Additionally, researchers have highlighted that those involved in team sports require a diverse range of qualities (e.g., Cone [9]; Di Salvo et al., [10]; Little and Williams [11]; Oliver et al. [12]), speaking to the nuance and complexity of terms such as athleticism, athletic, and athlete.

High-level performance in a specific sport is very nuanced, and therefore a single expression or assessment of athleticism (e.g., jump height) is unrealistic. Despite several attempts by various researchers, there is no general consensus for athleticism [13, 14]. Lloyd et al. (p. 1491 [15]) defined it as the “ability to repeatedly perform a range of movements with precision and confidence in a variety of environments, which require competent levels of motor skills, strength, power, speed, agility, balance, coordination, and endurance”. Turner et al. [14] recommended that multidisciplinary coaching staff in team sport settings align on a holistic indication of an individual’s athleticism to help inform selection and development. However, in team sport contexts, determining whether a specific participant is a good ‘athlete’ usually reflects the relationship between performance attributes and positive outcomes [16]—not an individual’s need to perform a specific athletic task well in isolation (i.e., sprinting, jumping). In other words, the focus is on whether an individual can use a specific athletic attribute to positively influence outcomes (i.e., speed of transition from defense to attack, goal threat from set pieces), usually regardless of the attribute’s relevance for future long-term development. When proposing the use of a framework to grade/score athleticism, Turner et al. [14] argued that although an individual’s jump height may have value to some stakeholders (e.g., athletic development staff), scores in isolation may not prove overly helpful for others. A coach, for example, is perhaps more concerned about how this attribute positively influences specific match outcomes (e.g., winning a header in soccer).

The broader relevance of an individual’s athleticism compared to his/her/their teammates may be equally (or more) important. Athleticism for a team sport player is known to improve outcomes at elite levels and is often vital during pivotal moments of a game (e.g., Di Salvo et al. [10]; Little and Williams5 [11]; Oliver et al. [12]). However, it is important to note that the purpose of individual development in team sports is not solely to produce athletes, but instead to promote the individual’s acquisition of a set of robust technical and tactical sport-specific skills that allows him/her/them to meet training and competition demands. It is also worth recognizing that sport in general also offers the opportunity to develop the individual beyond the sport (i.e., character traits, confidence, competence, social skills) [17]. From this perspective, an athlete (i.e., an individual with athleticism) is valued based on the extent to which these qualities relate to the execution of key tasks and performance demands. When using ‘athlete’ to describe participants in a study, we therefore encourage researchers and practitioners to provide sufficient information to capture their level of competition, while also explaining what they mean by using different terms such as athlete.

## Categorization of Individuals in Team Sports: Athletes or Players?

Adding to the terminological confusion in sport is the definition of ‘player’. For example, the categorization of ‘player’ is commonly used in both applied and research settings from team sports to individual sports to sedentary games [18–21]. Specifically, practitioners and researchers have been shown to refer to some individual sport participants as players [19], despite this not always being a team activity (i.e., tennis players). This most likely relates to the verb used to describe the action of the sport (e.g., someone ‘plays’ tennis, but someone who cycles, ‘rides/cycles’, and would be considered a ‘rider/cyclist’ not a ‘player’). Moreover, people involved in team sports, such as coaches, spectators, and journalists, regularly use ‘player’ [22] to categorize participants in some sports (but not all). Additionally, some individual and team sport research has referred to both athletes and players simultaneously [18, 19,, 21], while other researchers (especially in genetics; McAuley et al. [23]) describe their participants as athletes instead of specific sport players.

Terminological blurriness is further evident in more sedentary sports. For instance, someone who participates in lower physical exertion activities (e.g., darts, snooker, chess) is also commonly known as a ‘player’ (e.g., darts player). Conversely, those who compete in other types of lower physical exertion activities are also (sometimes) called ‘athletes’. In e-sports, for instance, competitive and professional e-sport participants are often referred to as athletes, despite the activities being mostly sedentary [24]. However, consistency in this sporting categorization does not exist and some argue against e-sports’ place in ‘sport’ altogether [20].

It could be argued that those who participate in certain sports should perhaps be considered as players, instead of athletes, as the root of the term player is ‘play’. For instance, while some team sport individuals will be athletic, the term player may better capture someone who plays a game, often with fellow players in a dynamic and open setting, whereas an athlete may better capture someone whose competitive success is based to a greater extent on their athleticism (e.g., track and field) rather than their tactical, technical, psychological, and social abilities [25, 26]. Considering the holistic methodology that is often deployed by coaches to aid development [27, 28], this terminology may align better with this modern approach.

## Exploring Alignment with Theoretical Models of Youth Development

Various theoretical models of development in sport have described participants as either ‘athlete’, ‘talent’, or ‘player’. For instance, some of the most well-known models include the Long-Term *Athlete* Development model [29] and the Foundations, *Talent*, Elite, and Mastery framework [30]. Other approaches have focused on the description of developmental processes and the prediction of expertise (see Bruner et al. [31] and Coutinho et al. [32] for reviews). Alongside the Developmental Model of Sports Participation (Côté [33]), these are perhaps the most recognized and implemented models across sport governing bodies by policy makers and within sport organizations [8, 34].

Researchers have noted the lack of clarity around terms such as athlete and player in such models. Take, for example, Lloyd et al.’s [35] distinction between the terms ‘athlete’ and ‘talent’ in reference to models of youth development. In addition, some organizations have made adaptations to the terminology used in these theoretical models when applying them to their sport (e.g., The England Football Association and Canada Soccer’s implementation of a revised Long-Term Athlete Development model, referred to as the Long-Term Player Development model). Whilst it may be tempting to simply replace ‘athlete’ with ‘player’ in development models within team sports, these words may not be synonymous. Furthermore, differences in these terms could be particularly important at younger ages considering the positive association between an early profile of multisport engagement with success at adulthood (e.g., Barth et al. [36]; Güllich et al. [37]).

During childhood and preadolescence (i.e., up until approximately age 12 years), it is generally recommended that youth engage in a diverse range of sports and activities characterized by high levels of unstructured play that promote fun and social interaction to improve fundamental movement competency [38]. Later in development (e.g., after peak height velocity in some sports), youth enter more stable stages of development and should be physically and mentally ready to focus on one or two sports. This usually involves engaging in higher amounts of structured activity and deliberate practice in order to achieve expertise (e.g., the ‘investment years’ in the Developmental Model of Sports Participation; Côté and Vierimaa [38]). In many sports, a shift to more advanced elements of physical, psychological, and cognitive development occurs, often as these elements relate to competitive success.

From this perspective, ‘athlete’ and ‘player’ become anchors for the focus of development during these phases of the athlete pathway (i.e., developing elements of athleticism during early development and improving competitive play during later phases). More difficult to integrate is ‘talent’. Indeed, researchers and coaches have struggled to clarify what this word means in both scientific and practical settings. For example, Australia’s Foundations, Talent, Elite, and Mastery framework describes the period of development between early and late adolescence as the ‘talent phase’, whereby youth demonstrate their ‘potential’ (another unclear and inconsistently used word) and later have their ‘talent’ verified. However, this usage suggests ‘talent’ is not relevant at other times/stages in the athlete’s development and/or that ‘talent identification’ is appropriate during this specific phase of the pathway. More recent conceptualizations (e.g., Baker et al. [39]) position ‘talent’ as originating in innate biological structures that evolve through their interaction with environmental variables throughout development. From this perspective, it is not possible to separate a person into his/her/their ‘talent(s)’ versus his/her/their experiences because both are inextricably linked. However, this does not mean we should discard talent as a concept; rather, it simply means that we should use it in proper ways [40].

## Contextual and Methodological Considerations

While a global approach to the terms used in competitive sport across different stages of development may seem beneficial, it is important to consider the contextual and methodological influences that could explain the terminological confusion often seen in sport settings. For instance, the sociocultural norms of diverse communities are important considerations for researchers and practitioners when interpreting relevant research findings and implementing them into applied settings. As an example, how the term athlete is perceived in North America (e.g., Canada) may differ from how it is understood in Europe (e.g., the UK). Indeed, although many of the highly cited English-speaking researchers in the field of youth sport typically derive from these regions [41], they often use different terms to define developmental models and pathways for youth in sport.

In some instances, researchers use the word athlete as a homogeneous term to describe *all* youth who are engaged in sport (see Baker et al. [40]; Bruner et al. [31]; Varghese et al. [42] for reviews). For instance, the Personal Assets Framework is commonly referred to as an athlete development model (e.g., Côté et al. [43]; Kelly et al. [44]), which aims to develop youth across *all* participation levels, and focuses on characteristics beyond physical/athletic competencies. In comparison, other researchers use the term athlete development to describe the development of physical/athletic qualities in youth sport settings (e.g., Balyi et al. [29]), whereas talent development is more commonly used to define more holistic pathways for youth in sport (e.g., Bailey and Collins [45]; Coutinho et al. [32]; Mills et al. [46]). Based on these factors, generalizing terms could have consequences for translating research into applied practice, as the reader and/or end user may have different interpretations of what specific terms mean based on his/her/their regional norms, which could lead to poor global implementation.

The potential impact of sociocultural factors on terminology can be exemplified by the differences between individualistic (i.e., communities in which individuals strive for self-realization) and collectivist (i.e., communities in which individuals prioritize the needs of the group rather than the individual) cultures [47]. In the context of sport, considerations regarding an athlete, talent, or player in an individualistic talent development pathway may be completely different to those of a collectivistic pathway. As an example, Brown et al. (in press) [48] showed how a sample of British South Asian cricketers cited a range of examples of how their actions would be perceived as respectful in their own culture (e.g., staying away from alcohol during team social activities, avoiding eye contact, and not challenging the coach or voicing their opinion), but potentially disrespectful in White British culture. Moreover, a systematic review of longitudinal talent identification and development literature highlighted that almost all research in this discipline written in English had been conducted in and by authors from individualistic nations [41], which is likely similar across other sport science disciplines. As such, it is plausible that the discourse of ‘best practice’ coupled with the language used in sport prioritizes individualistic approaches and, thus, potentially contradicts and marginalizes collectivistic behaviors, developmental strategies, and terminologies. It will be important to expand the knowledge on the methods and practices of more collectivist cultures to create a more detailed and diverse understanding of the language used in those contexts.

It is also important to highlight that many leading journals, empirical studies, and reviews are written in English. Whilst it could be considered the global language, it is not the native language of a large proportion of the world. This makes it even more important to ensure that the language used in sport is understandable and transferrable to those who are not native English speakers. We should not see this as a barrier, but rather should instead embrace this cultural diversity to broaden our horizons and work towards a common understanding. Using a common language could help dispel negative stereotypes and personal biases about different groups, as well as support cultural diversity through respecting and learning about ways of being that are not necessarily our own. Building common terminology could facilitate these interactions with others so we can build bridges across cultures. However, it should be noted that this will be a difficult task owing to the variety of languages spoken, adding further difficulty to creating global terminologies. This should not, however, be an excuse to use vague language, and efforts should be made to clearly define key terminologies within research and practice.

The complexity of applying a singular term and definition in research settings could also have implications on the uptake of knowledge and knowledge application. If the end user misinterprets key terminology, it could lead to actions and judgments that are misguided. Therefore, a knowledge mobilization approach is encouraged when designing methods to define key terminologies in the field of sport. The aim of knowledge mobilization is to encourage researchers and practitioners to work collaboratively to support evidence-informed policies and practices [49]. In the context of language, the co-design of consistent words and definitions will help with greater transparency and alignment between research and practice (e.g., shared mental model), whereby the terms used in research are moved from the study (e.g., article, book, conference, laboratory) into the hands of people and organizations who can put them to practical use [50]. Involving practitioners in the creation of definitions will also improve clarity, as researchers can be over-confident regarding the transferability of their results to real-life settings, whereas practitioners may not have the skills or resources (e.g., financial, human, time) to translate research results into practice.

Based on the contextual (e.g., sociocultural norms, individualism and collectivism, native language, game/sport type) and methodological (e.g., embrace diversity and diverse perspectives, prioritize collaboration and co-design, use clear and consistent terminology, practice knowledge mobilization) considerations presented above, it is unlikely there will be universally accepted (and utilized) definitions of athlete, talent, or player in the immediate future. It is hoped this discussion encourages stakeholders to take initial steps toward more concrete understandings and alignment with terms used in athlete development contexts. At the very least, researchers should be explicit in their rationale and relay unbiased balanced conclusions to allow the audience to draw their own interpretations. As illustrated by McAuley et al. [3], this should include transparency in the elements that are relevant to the categorization of sport participants, such as: (a) age; (b) competition level; (c) league status; (d) sex; (e) international ranking; (f) nationality; (g) province/state; (h) sport; and (i) success/achievements.

## Conclusions

Achieving generalizable definitions of specific sporting cohorts may be difficult because of contextual and methodological interpretations. However, clearer definitional boundaries for which terms can be used and operationalized are of importance in future research to better reflect contemporary academic thought and drive future athletic and technical development planning. We propose a conceptual distinction between terms used to describe different types of sport participants at different stages of development. Distinguishing between these terms may better capture differences in the activities current frameworks of development suggest youth should be engaged in at different developmental timepoints. In addition, discussions of what different words mean in different contexts encourage a more thorough understanding of the power of language in coaching and athlete development settings.
